# Evoked potentials in stroke rehabilitation: current applications, emerging technologies, and future directions

**DOI:** 10.3389/fnins.2026.1758767

**Published:** 2026-02-18

**Authors:** Zhe Wang, Xiaolin Liu, Jingyang Xie, Yujun Lin

**Affiliations:** 1 Laboratory of Neuroelectrophysiology, Yantai Yantaishan Hospital, Yantai, China; 2 Department of Neurology, Zibo 148 Hospital, China RongTong Medical Healthcare Group Co. Ltd., Zibo, China; 3 Laboratory of Electrophysiology, Yantai Yantaishan Hospital, Yantai, China

**Keywords:** ERPs, evoked potentials, MEPs, SEPs, stroke

## Abstract

Evoked potentials (EPs) are increasingly explored as objective neurophysiological biomarkers to complement scale-based assessment in stroke rehabilitation. This narrative review summarizes current evidence on the use of somatosensory evoked potentials (SEPs), motor evoked potentials (MEPs), and event-related potentials (ERPs) for monitoring recovery and guiding therapy. We first outline the physiological basis and stroke-relevant features of each modality, then synthesize data on how EP measures relate to motor, sensory, balance, cognitive and language outcomes, with particular emphasis on longitudinal changes during rehabilitation and responses to specific interventions, including neuromuscular electrical stimulation, robot-assisted training and non-invasive brain stimulation. Emerging applications such as perturbation-evoked cortical responses for postural control, EP-based brain–computer interfaces and EP-guided or closed-loop neuromodulation are discussed, together with advances in high-density recordings, connectivity analysis, and machine-learning–based multimodal prediction models. Finally, we highlight key methodological and practical challenges—protocol heterogeneity, small single-center studies, limited trial evidence, feasibility constraints and gaps in clinical integration—and propose priorities for standardization and translational research. Overall, EPs hold substantial promise as pathway-specific, temporally precise biomarkers to enable more mechanism-informed and individualized stroke rehabilitation monitoring.

## 1 Introduction

Stroke remains a leading cause of adult disability worldwide and is a major contributor to long-term functional dependence and reduced quality of life (GBD 2019 Stroke Collaborators, 2021; Murray et al., 2012). Despite advances in acute reperfusion therapies and organized stroke unit care, a substantial proportion of survivors are left with persistent motor, sensory, cognitive, and language impairments that require prolonged rehabilitation. In routine practice, the effectiveness of rehabilitation is primarily evaluated using clinical rating scales such as the Fugl–Meyer Assessment, the modified Rankin Scale, the Barthel Index, and various cognitive and language batteries (Gladstone et al., 2002; McGill et al., 2022). While these instruments are indispensable, they are inherently subjective, may suffer from ceiling and floor effects, and often lack sensitivity to early or subtle neurophysiological changes that precede overt functional gains.

In this context, neurophysiological biomarkers have attracted growing interest as objective, quantitative tools to complement clinical assessment. Among them, evoked potentials (EPs) represent a family of electrophysiological techniques that capture the brain’s time-locked responses to controlled sensory, motor, or cognitive events. Because EPs directly probe the integrity and plasticity of specific neural pathways and cortical networks, they are well suited to track lesion impact, spontaneous recovery, and training-induced reorganization across the continuum of stroke care (Burghaus et al., 2008; Passmore et al., 2014). Over the past decades, a substantial body of evidence has demonstrated that EP measures—such as motor evoked potentials (MEPs) elicited by transcranial magnetic stimulation, somatosensory evoked potentials (SEPs) following peripheral nerve stimulation, and event-related potentials (ERPs) like the P300 during cognitive tasks—are associated with motor outcome, sensory restitution, and cognitive recovery after stroke (Spampinato et al., 2023). In many studies, the presence, amplitude, or latency of these responses has been used to predict prognosis or to stratify patients for specific interventions.

However, most existing work has focused on EPs as prognostic markers at one or a few discrete time points, typically in the acute or early subacute phase (Fuhr et al., 2001; Hardmeier et al., 2022; Oertel et al., 2023). In contrast, the potential of EPs as dynamic monitoring tools throughout the rehabilitation process—capturing evolving cortical excitability, pathway conduction, and cognitive processing—has received comparatively less structured attention. For clinicians, the key practical questions are not only “What does this patient’s EP tell us about eventual recovery?” but also “How do these EP measures change with ongoing therapy?” “Can they signal whether a given rehabilitation strategy is effective or needs adjustment?” and “Could EP-guided, individualized rehabilitation improve outcomes compared with conventional, scale-driven approaches?”

At the same time, technological progress in electrophysiology and data analysis is rapidly expanding what can be measured and inferred from EP signals (van de Leur et al., 2020). High-density recordings, advanced source localization, connectivity metrics, and machine-learning–based feature extraction allow EPs to move beyond simple peak-to-peak amplitudes and latencies toward richer, network-level and multivariate descriptors of brain function. EPs are also increasingly integrated into brain–computer interface (BCI) paradigms and closed-loop neuromodulation systems, where they serve not only as outcome measures but also as control signals that directly shape therapeutic stimulation or task difficulty (Belkacem et al., 2023).

Despite these promising developments, important challenges remain. Methodological heterogeneity in stimulation protocols, recording montages, and data processing pipelines complicates cross-study comparisons and limits the derivation of robust normative and pathological thresholds. Many studies are single-center, with small samples and short follow-up periods, and few have been designed to evaluate EP-driven decision-making or EP-based endpoints in randomized controlled trials. Practical barriers—including equipment costs, time constraints in busy rehabilitation units, and the need for specialized expertise—also hinder the widespread adoption of EPs in everyday clinical practice.

**FIGURE 1:**
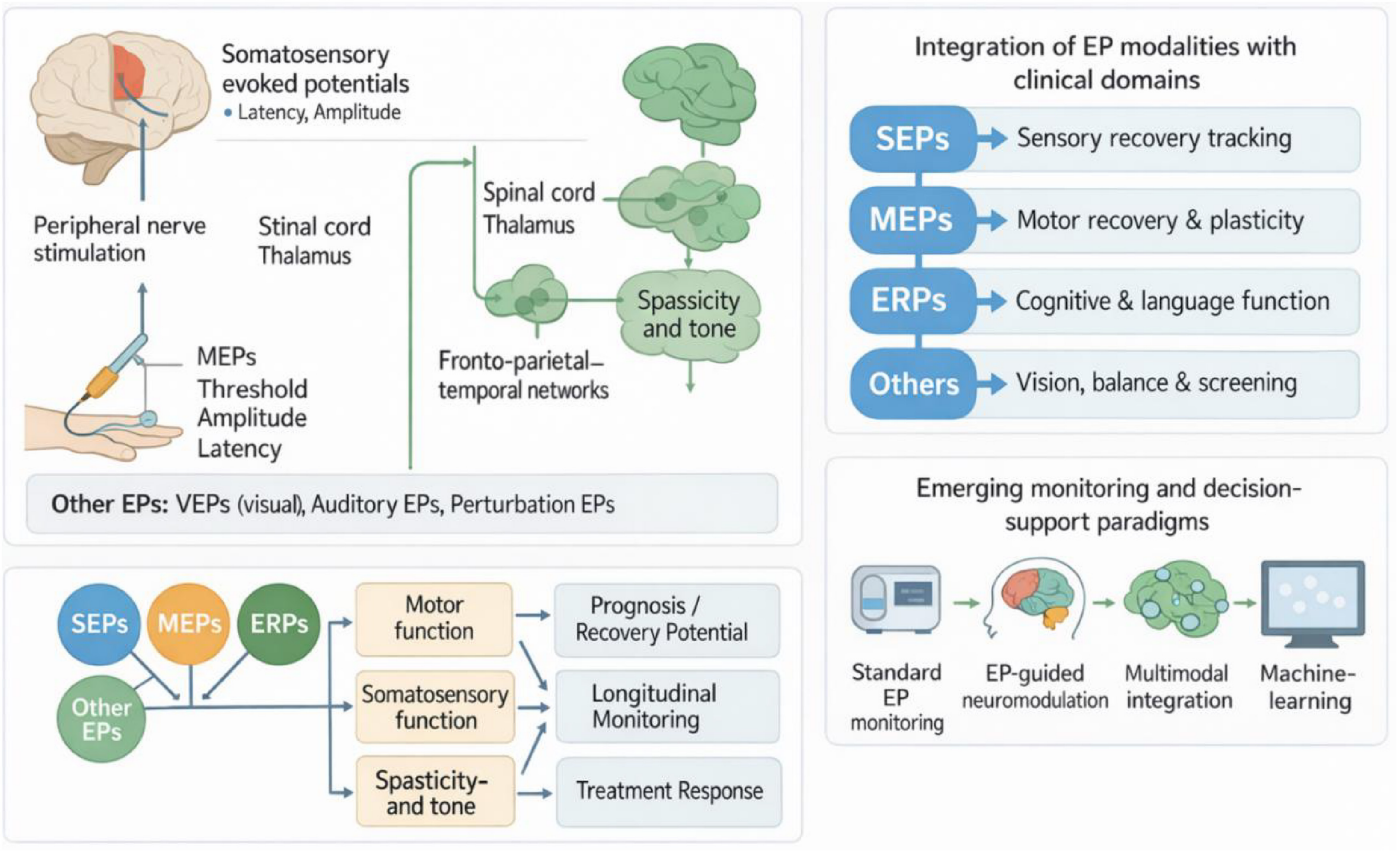
Conceptual framework of evoked potentials in stroke rehabilitation monitoring. EP, evoked potential; SEP, somatosensory evoked potential; MEP, motor evoked potential; ERP, event-related potential; VEP, visual evoked potential; BCI, brain–computer interface.

Against this background, the present review aims to provide a clinically oriented overview of the innovative advances and remaining challenges of evoked potentials in stroke rehabilitation monitoring. First, we summarize the basic principles and stroke-relevant features of the main EP modalities used in rehabilitation—somatosensory, motor, and cognitive event-related potentials—and briefly introduce other emerging approaches. We then discuss how these modalities have been applied to monitor motor, sensory, balance, and cognitive recovery, including their longitudinal dynamics during specific rehabilitation interventions. Next, we highlight emerging technologies such as EP-guided neuromodulation, BCI-based rehabilitation, and multimodal integration with imaging and wearable sensors. Finally, we analyze methodological and translational barriers and outline future directions toward the incorporation of EP-based biomarkers into individualized, mechanism-informed stroke rehabilitation pathways (Figure 1).

## 2 Methods of the review

This article is a narrative review that aims to provide a clinically oriented synthesis of evoked potentials (EPs) for stroke rehabilitation monitoring, rather than a formal systematic review. To enhance transparency and reproducibility, we report the core elements of our evidence identification and synthesis process, guided by established recommendations for narrative reviews (Baethge et al., 2019).

We searched PubMed/MEDLINE and Web of Science Core Collection for English-language publications (until 2026). Search terms were constructed by combining stroke and rehabilitation concepts with EP modalities and related technologies, including: (“stroke,” “cerebrovascular,” “post-stroke,” “rehabilitation,” “recovery,” “neurorehabilitation,” “evoked potential,” “somatosensory evoked potential,” “SEP,” “motor evoked potential,” “MEP,” “event-related potential,” “ERP,” “TMS,” “EEG”) and, for emerging applications, (“brain–computer interface,” “BCI,” “closed-loop,” “neuromodulation”). Reference lists of key reviews and seminal studies were also hand-searched to identify additional relevant reports.

We included studies that investigated EP measures (SEPs, MEPs, ERPs or related paradigms) in adult stroke populations and reported associations with functional outcomes, longitudinal changes during rehabilitation, or responses to specific interventions (e.g., task-oriented training, robotic therapy, neuromuscular stimulation, non-invasive brain stimulation, or BCI-based rehabilitation). We excluded purely methodological papers without stroke relevance, and reports lacking sufficient methodological description to interpret EP acquisition/analysis. Title/abstract screening and full-text eligibility assessment were performed by two authors, with disagreements resolved by consensus.

Given the heterogeneity of EP protocols and outcome measures, we synthesized evidence qualitatively, prioritizing: (i) convergent findings replicated across cohorts; (ii) clinically interpretable EP parameters (presence/absence, latency, amplitude, thresholds); and (iii) longitudinal/within-subject designs where available. For emerging technologies (high-density recordings, source analysis, connectivity metrics, machine learning, and multimodal fusion), we focused on proof-of-concept feasibility, sources of variability, and translational considerations relevant to routine rehabilitation practice.

## 3 Overview of evoked potentials relevant to stroke rehabilitation

Evoked potentials comprise a family of electrophysiological techniques in which neural activity is measured in response to controlled stimuli or perturbations. In the context of stroke rehabilitation, three EP domains are of particular relevance: somatosensory evoked potentials (SEPs), motor evoked potentials (MEPs), and event-related potentials (ERPs) associated with cognitive processing. Each modality interrogates different neural pathways, offers distinct advantages and limitations, and is suited to specific clinical questions.

### 3.1 Somatosensory evoked potentials (SEPs)

Somatosensory evoked potentials are elicited by repetitive electrical stimulation of peripheral nerves—most commonly the median nerve at the wrist for upper limb assessment and the tibial or peroneal nerve at the ankle or knee for lower limb evaluation (Passmore et al., 2014). The resulting cortical responses are recorded from scalp electrodes placed over the somatosensory cortex, typically referenced to midline or contralateral sites. In clinical practice, the early cortical SEP components (such as N20 and P25 following median nerve stimulation) are of particular interest, as they reflect conduction and synaptic transmission along the dorsal column–medial lemniscus pathway and primary somatosensory cortex (Toleikis et al., 2024).

In stroke patients, SEPs provide objective information on the integrity of somatosensory pathways and cortical processing that may not be fully captured by bedside sensory examination. The presence or absence of cortical SEPs, as well as changes in their amplitude and latency, have been associated with the severity of sensory deficits, functional recovery of hand and limb use, and overall outcome (Fuseya et al., 2025). Moreover, SEPs have been employed to monitor the neurophysiological effects of various interventions, such as peripheral electrical stimulation, task-specific training, and robotic-assisted therapy, under the assumption that improvements in SEP parameters reflect adaptive plasticity within the sensory system. SEPs thus occupy a central role in efforts to track sensory restoration and to understand how sensory inputs shape motor relearning during rehabilitation.

### 3.2 Motor evoked potentials (MEPs)

Motor evoked potentials are most commonly elicited using transcranial magnetic stimulation applied over the primary motor cortex. A brief, high-intensity magnetic pulse induces a transient electric field in the underlying cortical tissue, depolarizing pyramidal neurons and generating descending volleys along the corticospinal tract. The resulting compound muscle action potentials are recorded from target muscles—such as the abductor pollicis brevis or tibialis anterior—using surface electromyography (Deletis and Sala, 2008; Spampinato et al., 2023).

Motor evoked potentials provide a direct, noninvasive probe of corticospinal excitability and conduction. Key parameters include the presence or absence of a response at a given stimulus intensity, the resting motor threshold, the peak-to-peak amplitude, and the latency of the response. In the stroke context, the detectability and characteristics of MEPs from paretic muscles have been strongly linked to motor outcome. Patients with preserved or recoverable MEPs in the affected limb generally have a higher probability of meaningful motor recovery, whereas those without MEPs often exhibit more severe and persistent deficits (Shanks and Byblow, 2025). Beyond this prognostic role, MEPs can be repeatedly measured during rehabilitation to track changes in corticospinal excitability over time and in response to interventions such as intensive task practice, constraint-induced movement therapy, robotic training, and non-invasive brain stimulation (e.g., repetitive TMS or transcranial direct current stimulation) (Tatemoto et al., 2017). As such, MEPs are prime candidate biomarkers for monitoring motor system plasticity and for tailoring rehabilitation strategies to individual neurophysiological profiles.

### 3.3 Event-related potentials (ERPs)

Event-related potentials are derived from electroencephalographic recordings time-locked to cognitive, sensory, or motor events. Unlike SEPs and MEPs, which chiefly reflect conduction along relatively well-defined sensorimotor pathways, ERPs capture the summed postsynaptic activity of large neuronal populations engaged in information processing (Sutton et al., 1965). ERP paradigms typically involve structured tasks such as oddball paradigms, go/no-go tasks, or language comprehension tasks, and focus on specific components (e.g., N1, P2, N2, P300, N400) that are thought to index processes like attention allocation, stimulus discrimination, working memory updating, or semantic integration (Sur and Sinha, 2009; Woodman, 2010).

In stroke rehabilitation, ERPs have gained attention mainly as tools to assess and monitor cognitive and language functions, which are often under-recognized but significantly affect functional outcome and participation. The P300 component, for example, is widely used as a marker of attention and working memory updating. Prolonged P300 latency and reduced amplitude have been reported in stroke survivors with cognitive impairment and may improve with cognitive rehabilitation, suggesting their potential role as objective indicators of treatment response. ERPs have also been used to evaluate residual cognitive processing in patients with severe motor impairments and to support communication via P300-based brain–computer interfaces, thereby blending assessment and intervention within the same paradigm (Li et al., 2021; Polich and Criado, 2006; Polich, 2007).

Beyond P300, other ERP components may inform specific domains: early sensory components can indicate the integrity of auditory or visual processing, while later components related to conflict monitoring, error processing, or language comprehension may provide more fine-grained insight into higher-order dysfunctions. However, ERP acquisition and interpretation can be more demanding than basic sensory or motor EPs, as they require task engagement, careful control of stimulus properties, and more complex signal processing (Poskotinova et al., 2023).

### 3.4 Other modalities and multimodal approaches

In addition to SEPs, MEPs, and ERPs, other EP modalities may contribute to stroke rehabilitation monitoring in selected contexts. Visual evoked potentials and auditory brainstem responses, for instance, can be used to evaluate visual and auditory pathway integrity in patients with corresponding deficits, potentially informing rehabilitation strategies in domains such as reading, communication, and spatial orientation (Kashani et al., 2021). Long-latency responses and polysensory paradigms are being explored to assess integration across sensory systems and to probe higher-order networks involved in body representation and spatial neglect (Lieberman, 2008).

Importantly, EPs do not exist in isolation. In contemporary neurorehabilitation research, EPs are increasingly combined with structural and functional imaging, quantitative kinematic and kinetic measurements, and data from wearable sensors and digital health technologies. Such multimodal approaches aim to provide a more comprehensive picture of the recovering brain and body, leveraging the strengths of each technique—temporal precision in EPs, spatial resolution in imaging, and ecological validity in wearable monitoring—to better understand and guide rehabilitation. Having outlined the main EP modalities relevant to stroke rehabilitation, the following sections examine in more detail how these techniques have been applied to monitor recovery in specific functional domains, summarize current evidence for their clinical utility, and discuss emerging technologies and implementation challenges (Table 1).

**TABLE 1:** Summary of main evoked potential modalities used in stroke rehabilitation.

Modality	Typical stimulus/paradigm	Main target	Key metrics	Main rehabilitation applications
SEPs	Peripheral nerve stimulation (e.g., median)	Dorsal column–medial lemniscus	Presence/absence; latency; amplitude	Sensory pathway integrity; sensory recovery prognosis; contribution to balance/motor
MEPs	Single-pulse TMS over M1 with EMG	Corticospinal tract; spinal motor units	Motor threshold; MEP amplitude; latency	Motor recovery prognosis; recovery-potential stratification; monitoring training/neuromodulation
ERPs	Cognitive tasks (oddball, go/no-go, language)	Fronto-parietal and temporal networks	Component latencies and amplitudes (e.g., P300, N400)	Cognitive and language impairment detection; monitoring cognitive/aphasia rehab; BCI control
VEPs	Pattern-reversal or flash visual stimuli	Optic pathways; visual cortex	P100 latency; P100 amplitude	Visual pathway screening; visual contribution to reading, navigation and balance
Other EPs (auditory/postural)	Clicks/tone bursts; postural perturbations	Auditory pathways; postural networks	Brainstem wave latencies; cortical/auditory or postural responses	Auditory contribution to communication/cognition; mechanistic balance and fall research

## 4 Evoked potentials for monitoring motor recovery

Motor recovery is a central goal of stroke rehabilitation, and MEPs have become the most widely studied EP modality in this context. While early work emphasized the prognostic value of MEPs in predicting eventual motor outcome, there is growing interest in how MEPs and related measures can be used to monitor dynamic changes in corticospinal function during rehabilitation and to guide individualized treatment planning.

### 4.1 Baseline MEPs and early prognosis

In the acute and early subacute phases after stroke, the presence or absence of MEPs in muscles of the paretic limb provides a powerful indicator of corticospinal tract integrity. Patients in whom MEPs can be elicited from the affected hand or leg—either at rest or with mild voluntary activation—are much more likely to regain useful motor function than those with absent responses. Baseline MEP parameters such as motor threshold, response amplitude, and central motor conduction time have repeatedly been associated with later performance on clinical scales including the Fugl–Meyer Assessment and functional independence measures.

From a monitoring perspective, these baseline assessments serve as the starting point for tracking recovery. In patients with preserved MEPs, serial measurements can reveal whether corticospinal excitability is increasing as expected with rehabilitation. In patients initially lacking MEPs, the later emergence of responses may signal re-establishment of corticospinal conduction or recruitment of alternative descending pathways. Thus, even in their simplest form, baseline and follow-up MEPs provide an objective framework for categorizing patients into different recovery trajectories (Araç et al., 1994; Hendricks et al., 2002).

### 4.2 Longitudinal changes in MEPs during rehabilitation

A key advantage of MEPs is that they can be safely repeated at multiple time points during the rehabilitation course. Longitudinal studies have shown that, in many patients, MEP amplitudes increase and motor thresholds decrease over weeks to months of training, reflecting heightened corticospinal excitability and improved synaptic efficacy within the motor network. These neurophysiological changes often parallel improvements in voluntary strength, dexterity, and functional use of the affected limbs.

In some cohorts, early increases in MEP amplitude during the first few weeks of therapy have been associated with better motor outcomes at later follow-ups, suggesting that MEP dynamics may provide an early readout of “training responsiveness.” Conversely, a lack of change or deterioration in MEP parameters despite intensive rehabilitation may indicate limited potential for further recovery, prompting clinicians to adjust goals, modify strategies, or prioritize compensatory approaches. Importantly, MEP measures can detect subtle changes in corticospinal function even when bedside strength testing or global functional scales show minimal or delayed improvement, offering a more sensitive window into underlying plasticity (Ackerley et al., 2024; Yen et al., 2023).

### 4.3 Interaction with specific rehabilitation interventions

Motor evoked potentials have been used to monitor neurophysiological responses to a range of motor rehabilitation interventions. In constraint-induced movement therapy, for example, increased MEP amplitudes and expanded cortical representations of the paretic hand have been observed in responders, reflecting use-dependent reorganization of the ipsilesional motor cortex. Robotic-assisted training and intensive task-specific practice have similarly been associated with MEP changes that correlate with gains in motor performance (Sawaki et al., 2014).

Non-invasive brain stimulation techniques, particularly repetitive transcranial magnetic stimulation and transcranial direct current stimulation, have a direct impact on cortical excitability. MEPs are therefore natural biomarkers to assess whether these interventions are achieving their intended physiological effects. In protocols aiming to upregulate ipsilesional excitability, an increase in MEP amplitude or a reduction in motor threshold after stimulation indicates successful facilitation of the targeted motor areas. In inhibitory protocols targeting the contralesional hemisphere, changes in interhemispheric balance inferred from bilateral MEP recordings can help confirm that excessive transcallosal inhibition is being attenuated. By repeatedly assessing MEPs before, during, and after such interventions, clinicians and researchers can establish dose–response relationships and refine stimulation parameters to maximize beneficial plasticity.

### 4.4 Stratification and clinical algorithms based on MEP profiles

Beyond monitoring, MEPs can support decision-making by stratifying patients according to their residual corticospinal capacity. Prognostic algorithms have been proposed in which the initial presence of MEPs, combined with early clinical scores and structural imaging markers such as corticospinal tract lesion load, is used to allocate patients to recovery potential categories. Those with preserved MEPs and mild to moderate deficits are expected to achieve substantial recovery and may be prioritized for intensive, task-oriented therapies. Patients with absent MEPs and extensive corticospinal damage are less likely to regain fine distal control and may benefit from greater emphasis on compensatory strategies, assistive devices, and training of the non-paretic limb (Bembenek et al., 2020).

Integrating this stratification with longitudinal monitoring strengthens its clinical utility. For example, in a patient with initially weak but present MEPs, a failure to show any neurophysiological improvement after a defined period of intensive rehabilitation may prompt reconsideration of the therapeutic plan. Conversely, the appearance of MEPs in a previously unresponsive limb can motivate continued high-intensity training and justify the allocation of time and resources. In this way, MEP-based profiles can move from being static prognostic labels to dynamic guides for personalized, responsive rehabilitation.

Prognostic frameworks increasingly combine early clinical severity with biomarkers of pathway integrity to improve prediction of functional recovery. A representative example is the PREP2 algorithm, which integrates early clinical assessment with transcranial magnetic stimulation-derived MEP status (and imaging when required) to predict upper-limb outcome after stroke (Stinear et al., 2017). Within such models, EPs primarily serve as pathway-integrity biomarkers-most notably, MEP presence/absence provides a neurophysiological readout of corticospinal tract viability that complements bedside impairment measures. Beyond early stratification, EPs also offer value as longitudinal monitoring biomarkers: repeated MEP assessments may capture within-person change in corticospinal excitability and help interpret recovery trajectory or training responsiveness when clinical scales lag behind.

## 5 SEPs and other EPs in sensory, balance, and spasticity monitoring

Although motor impairment often dominates the clinical picture after stroke, sensory deficits, balance disturbances, and abnormal muscle tone substantially contribute to disability and fall risk. Somatosensory evoked potentials and related EP measures provide valuable information about these domains, enabling more nuanced assessment and potentially informing targeted interventions.

### 5.1 Sensory recovery and SEP dynamics

Clinical sensory examination is inherently subjective and may be influenced by factors such as attention, fatigue, and communication ability. SEPs, in contrast, offer an objective assessment of the integrity and functional status of the somatosensory pathways. In patients with paresthesia, numbness, or proprioceptive loss, absent or markedly delayed cortical SEPs from the affected limb indicate significant pathway disruption, whereas preserved or only mildly abnormal SEPs suggest residual conduction that may support recovery (Fuseya et al., 2025).

Longitudinally, improvements in SEP amplitude and reductions in latency have been reported in association with sensory training, peripheral electrical stimulation, and combined sensorimotor interventions (Mijic et al., 2022). Such changes may precede or exceed the recovery detectable by bedside testing, suggesting that SEP monitoring can reveal early signs of sensory plasticity. From a clinical perspective, documenting SEP improvements can help validate the effectiveness of interventions that emphasize sensory re-education, tactile discrimination training, or proprioceptive exercises. Conversely, persistently absent SEPs despite adequate rehabilitation may identify patients for whom compensatory strategies and environmental adaptations should be prioritized.

### 5.2 Balance, postural control, and multimodal EP assessment

Balance control depends on the integration of visual, vestibular, and somatosensory inputs, as well as the generation of appropriate postural responses. Stroke-related deficits in any of these components can lead to instability and falls (Li et al., 2019). While clinical tests such as the Berg Balance Scale or Timed Up and Go test capture global performance, they do not dissect the underlying sensory and motor contributions (Herman et al., 2011; Meseguer-Henarejos et al., 2019).

Somatosensory evoked potentials and MEPs can both contribute to a more mechanistic assessment of balance problems. For example, delayed or reduced SEPs from lower limb nerves may signal impaired proprioceptive input from the ankles and knees, which in turn can compromise postural adjustments during standing and walking. Abnormal MEPs in antigravity muscles can reflect weakened or poorly coordinated motor output. Combining these measures with clinical balance tests may clarify whether a patient’s instability is predominantly driven by sensory, motor, or integrative deficits and thus suggest tailored interventions—for instance, focused proprioceptive training versus strength and coordination exercises (Hwang et al., 2016; Kim et al., 2020; Lee et al., 2015).

Evoked potentials can also be recorded in paradigms that involve unexpected perturbations or platform translations, allowing assessment of long-latency postural responses and their cortical components. Although such paradigms are more complex and currently confined mainly to research settings, they illustrate the potential of EPs to probe the neural control of balance beyond static measures (Yoon et al., 2021).

### 5.3 Spasticity and sensorimotor network function

Spasticity and other forms of abnormal muscle tone are common after upper motor neuron lesions and can severely limit function and comfort. Clinically, spasticity is usually rated with ordinal scales such as the Modified Ashworth Scale, which are susceptible to inter-rater variability and may not reflect underlying neurophysiology (Zurawski et al., 2019). EPs offer several avenues to interrogate sensorimotor network function relevant to spasticity.

Within the SEP domain, certain components, such as the N30 response arising from premotor and supplementary motor areas, have been linked to sensorimotor integration and proximal limb control. Alterations in these components have been associated with the severity of spasticity and motor impairment in some studies, suggesting that they might serve as markers of maladaptive cortical reorganization. Similarly, changes in MEP recruitment curves, intracortical inhibition and facilitation measures, and H-reflex parameters can all provide insight into imbalances between excitation and inhibition at cortical and spinal levels (Spampinato et al., 2023).

Although the use of EPs as routine spasticity monitoring tools is still exploratory, longitudinal assessments could, in principle, help evaluate the effects of interventions such as botulinum toxin injections, antispastic medications, intrathecal baclofen, or specific stretching and strengthening programs. Demonstrating normalization of EP indices in parallel with clinical tone reduction would strengthen the mechanistic rationale for these treatments and support their refinement (Montane et al., 2025; Otero-Luis et al., 2024).

## 6 Event-related potentials for cognitive and language rehabilitation monitoring

Cognitive and language deficits are frequent after stroke and have profound implications for independence, social participation, and quality of life (Mancuso et al., 2023). Yet they can be underdiagnosed, especially when overshadowed by motor impairment. Event-related potentials complement neuropsychological assessments by providing temporally precise, quantitative markers of specific cognitive operations. In rehabilitation, ERPs are increasingly explored as tools to detect impairments, monitor treatment response, and even facilitate communication in patients with severe motor limitations.

### 6.1 P300 and general cognitive function

Among ERP components, the P300 elicited in oddball paradigms is the most widely used in clinical research. It is typically interpreted as reflecting attentional resource allocation and working memory updating in response to infrequent, task-relevant stimuli. In stroke survivors, prolongation of P300 latency and reduction of amplitude have been associated with global cognitive impairment, including deficits in attention, processing speed, and executive function (Polich, 2007; Sur and Sinha, 2009).

During rehabilitation, repeated P300 assessments allow tracking of cognitive recovery. Improvements in P300 latency and amplitude have been reported following cognitive training programs, pharmacological interventions, and combined motor–cognitive therapies (Feng et al., 2025). Importantly, in some studies, ERP changes have emerged even when conventional cognitive scales showed modest or delayed improvements, indicating that ERPs may capture early neural plasticity underlying later behavioral gains. For patients with fluctuating performance due to fatigue or mood, ERPs provide a relatively task-robust complement to subjective reports and clinician impressions.

### 6.2 ERPs in domain-specific cognitive rehabilitation

Beyond global cognitive function, specific ERP paradigms can target particular domains. Go/no-go or flanker tasks yield components such as N2 and error-related negativity that index response inhibition and conflict monitoring, which are important for safe mobility and self-management (Donkers and van Boxtel, 2004; Liu et al., 2017). Working memory tasks probe sustained attention and manipulation of information, while visuospatial paradigms assess neglect-related processing biases. For each domain, characteristic ERP signatures can be used to identify deficits and track domain-specific rehabilitation.

In practice, this means that a stroke patient undergoing an attention training program could be evaluated with an oddball or continuous performance task ERP before and after the intervention. Changes in relevant components provide an objective indication of whether the neural systems supporting attention are becoming more efficient. Similarly, patients with executive dysfunction might show normalization of frontal ERP markers with targeted cognitive-behavioral therapy.

### 6.3 Language ERPs and aphasia rehabilitation

Language impairment (aphasia) is another area where ERPs hold promise. Components such as the N400, associated with semantic processing, and the P600, related to syntactic reanalysis, are sensitive to disruptions in language comprehension. In patients with aphasia, abnormal N400 or P600 responses to linguistic stimuli can be recorded even when behavioral responses are limited, providing insight into residual comprehension capacities (Seyednozadi et al., 2021).

During language rehabilitation, repeated ERP assessments can reveal whether training is restoring more normal patterns of cortical language processing. For example, an increase in N400 sensitivity to semantic incongruities or the emergence of a more distinct P600 may indicate improved integration and syntactic processing, even in the absence of dramatic changes in standard aphasia test scores. These measures could be especially informative in tailoring therapy intensity and content for individuals with severe expressive deficits who cannot easily demonstrate their understanding in verbal form (Angrilli et al., 2003; Russell-Meill et al., 2025).

### 6.4 ERPs in brain–computer interfaces for severe motor impairment

For patients with very severe motor deficits, including those with locked-in syndrome or minimal voluntary movement, ERPs are central to brain–computer interface applications. P300-based BCI systems, for instance, allow users to select letters or commands by attending to rare target stimuli in a visual or auditory matrix. In such systems, ERPs serve both as the control signal and as an implicit monitor of sustained attention and information processing capacity (McWeeny and Norton, 2020).

Within a rehabilitation framework, BCI use can itself be considered a form of cognitive training, and the quality of ERP responses over time may reflect improvements in attention, vigilance, and motivation. Although still a niche application, this intersection of assessment and intervention illustrates the broader potential of ERPs to support communication, autonomy, and participation in patients who are otherwise difficult to evaluate and treat (Table 2).

**TABLE 2:** Applications of evoked potentials across functional domains in stroke rehabilitation.

Functional domain	Main EP modality	Typical paradigms/metrics	Typical clinical applications/decisions
Motor function (upper/lower limb)	MEPs, SEPs	TMS–MEP (presence, threshold, amplitude, latency); limb SEPs	Motor prognosis; recovery-potential stratification; monitoring response to motor training and neuromodulation
Somatosensory function	SEPs	Median/tibial SEPs (latency, amplitude)	Characterizing sensory deficits; tracking sensory recovery; evaluating sensory re-education and peripheral stimulation
Balance and postural control	SEPs, MEPs, perturbation EPs	Lower-limb SEPs; MEPs in antigravity muscles; postural perturbation EPs (e.g., N1)	Mechanistic analysis of falls and instability; tailoring proprioceptive vs strength/coordination training; postural adaptation research
Spasticity and abnormal tone	MEPs, H-reflex, SEPs	MEP recruitment; intracortical inhibition/facilitation; H-reflex; N30	Complementing spasticity scales; assessing effects of botulinum toxin, antispastic drugs, intrathecal baclofen, stretching/strengthening
Cognition, language, communication	ERPs (P300, N2, N400, P600); ERPs in BCI	Oddball (P300); go/no-go (N2, error-related); semantic/sentence paradigms; P300 speller	Detecting and monitoring cognitive and language impairment; evaluating cognitive/aphasia rehabilitation; establishing alternative communication via BCI

## 7 Emerging technologies and multimodal monitoring paradigms

Technological advances in signal acquisition, processing, and integration are transforming the role of EPs in stroke rehabilitation from simple waveform measurements into components of rich, multimodal monitoring systems. Several developments are particularly relevant for future practice.

### 7.1 High-density recordings, source localization, and connectivity

Traditional EP recordings typically use a limited number of scalp electrodes focused on primary sensory or motor areas. High-density EEG systems, combined with advanced source localization algorithms, enable more detailed mapping of the cortical generators underlying EPs and ERPs. This allows researchers to move beyond a single peak recorded at a standard electrode to examine distributed networks and their coordinated activity. For example, high-density SEP recordings can reveal how activation spreads across primary and secondary somatosensory areas, parietal association cortex, and premotor regions during recovery. Similarly, ERP studies can characterize changes in functional connectivity between frontal and parietal regions as attention and executive functions improve. Connectivity metrics derived from EP data, such as coherence and phase synchronization, may offer sensitive markers of network-level reorganization that complement traditional amplitude and latency measures (Gindrat et al., 2015; Stoyell et al., 2021; Vorderwülbecke et al., 2021).

### 7.2 EP-guided neuromodulation and closed-loop stimulation

Another emerging trend is the use of EPs to guide and adapt neuromodulation strategies. In open-loop paradigms, baseline MEPs or SEPs are used to identify candidate targets and to set stimulation parameters. For instance, patients with low ipsilesional MEP amplitudes may receive facilitatory rTMS over the affected motor cortex, whereas those with excessive contralesional excitability may be targeted with inhibitory protocols (Luk et al., 2022). Closed-loop approaches go further by adjusting stimulation in real time based on ongoing EP or EEG signals. For example, stimulation can be delivered contingent on specific oscillatory phases or EP components that reflect optimal excitability states, potentially enhancing plasticity and reducing variability in response. In principle, such systems could monitor EPs during a therapy session and adapt stimulation intensity, timing, or site to maintain a desired neurophysiological state conducive to learning (Lo and Widge, 2017).

Although still in early stages, EP-guided neuromodulation represents a promising avenue for personalized rehabilitation, where objective neurophysiological feedback replaces fixed, one-size-fits-all stimulation protocols.

### 7.3 Integration with brain–computer interfaces and digital therapies

As digital and virtual rehabilitation therapies proliferate, there is increasing interest in embedding EP monitoring within these environments. For example, a patient using a virtual reality motor training system could simultaneously undergo MEP testing to quantify changes in corticospinal excitability across sessions. Similarly, cognitive training apps could be paired with ERP recording to track neurophysiological engagement and improvement (Bagce et al., 2013). In BCI-based rehabilitation, EPs are integral not only as control signals but also as metrics of training progress. EP characteristics such as signal-to-noise ratio, latency stability, and component amplitude can indicate whether the user is successfully engaging with the interface and whether task difficulty should be adjusted. The integration of EPs into these digital platforms opens the door to home-based, longitudinal monitoring that extends beyond the walls of the rehabilitation clinic.

### 7.4 Machine learning and multimodal data fusion

Finally, the rise of machine learning provides powerful tools for extracting informative patterns from high-dimensional EP data and combining them with other modalities. Rather than relying on a single peak amplitude or latency, machine learning models can integrate multiple EP features—across time, frequency, and space—alongside structural imaging, functional connectivity, clinical scores, and kinematic data. Such models can be trained to predict outcomes, classify patients into phenotypic subgroups, or forecast response to specific interventions. Importantly, they can also highlight which EP features carry the most predictive information, guiding future simplified protocols. Multimodal data fusion approaches may reveal combinations of EP, imaging, and behavioral markers that are more robust than any single measure, supporting the design of comprehensive, individualized monitoring frameworks (Koutsojannis and Chrysanthakopoulou, 2025; Rajendran et al., 2024).

Recent machine-learning studies have sought to improve early prediction of post-stroke outcomes and complications using multidimensional clinical data. For example, van der Gun et al. (2025) evaluated whether ML approaches can improve early prediction of upper-limb recovery, and Wu et al. (2025) developed an ML-based longitudinal model for early post-stroke fatigue prediction. In this context, EP-derived features (e.g., MEP presence/absence, amplitude/latency, or ERP indices of attention and processing) are attractive candidates because they provide mechanistic information about sensorimotor and cognitive network function that may not be fully captured by clinical scores alone. At the same time, prediction models and rehabilitation monitoring address different clinical questions: prediction aims to estimate likely outcome categories early, whereas longitudinal EP monitoring is designed to detect within-person change and treatment responsiveness over time. Making this distinction explicit helps avoid over-interpreting prediction models as prescriptive treatment rules and supports a complementary view in which EPs contribute both to early stratification and to trajectory-aware monitoring during rehabilitation.

## 8 Methodological and practical challenges

Despite the promise of EPs for stroke rehabilitation monitoring, several methodological and practical challenges must be addressed before their widespread clinical adoption.

### 8.1 Heterogeneity in protocols and outcome measures

Current EP studies in stroke vary widely in stimulation parameters, recording montages, signal processing pipelines, and outcome measures. For SEPs, differences in stimulation intensity, frequency, nerve selection, and averaging methods can lead to inconsistent peak identification and amplitude values. For MEPs, variations in coil type, stimulus intensity relative to motor threshold, muscle selection, and background contraction levels complicate comparisons across studies and centers. ERP paradigms differ in task design, stimulus parameters, and analysis windows. This heterogeneity hinders the establishment of normative databases, cut-off values for pathology, and standardized reporting of EP findings. It also limits the feasibility of meta-analyses and the generalization of results to different clinical settings. Developing consensus guidelines on EP acquisition and interpretation in stroke rehabilitation—analogous to those existing for intraoperative monitoring—would be an important step toward more uniform practice (Bembenek et al., 2012).

### 8.2 Study design limitations

Many EP studies in stroke rehabilitation are small, single-center, and observational in nature. Sample sizes are often insufficient to support robust multivariable modeling, and follow-up durations may be limited to a few weeks or months. Interventions are heterogeneous, and EP assessments are not always synchronized with standardized rehabilitation protocols or clinical endpoints. Moreover, relatively few randomized controlled trials have used EPs as primary or co-primary outcomes, and even fewer have incorporated EP measures into adaptive intervention algorithms. As a result, while there is substantial evidence that EPs correlate with recovery, there is less high-level evidence that EP-guided rehabilitation strategies improve outcomes beyond standard care. Addressing this gap will require well-designed, adequately powered trials that embed EP monitoring within clearly defined therapeutic pathways (Ackerley et al., 2024; Stinear, 2017).

### 8.3 Patient-related factors and feasibility

Evoked potential recording depends on patient cooperation to varying degrees. MEPs may require relaxation or specific muscle contractions; ERPs demand sustained attention and understanding of task instructions; SEPs may be influenced by arousal and medication. Cognitive impairment, aphasia, apathy, or fatigue can reduce data quality or necessitate simplified paradigms. In some patients with severe deficits or comorbidities, EP studies may not be feasible.

On the practical side, EP assessments take time and require trained personnel and specialized equipment. In busy rehabilitation units, it may be challenging to schedule repeated EP sessions around therapy appointments, medical procedures, and patient transport. Financial and reimbursement considerations further affect whether EP monitoring can be integrated into routine care.

### 8.4 Interpretation and clinical integration

Even when EP data are available, translating them into actionable clinical decisions is not always straightforward. For example, a modest increase in MEP amplitude could reflect genuine recovery, compensatory reorganization, or simply variability in recording conditions. Similarly, ERP changes may be influenced by practice effects or non-specific factors. Without clear thresholds or standardized decision rules, clinicians may be uncertain how to adjust rehabilitation plans based on EP findings.

To address this, EP reporting should ideally be linked to specific, practical recommendations—such as suggested therapy intensity, modality, or need for further diagnostic evaluation—rather than remaining purely descriptive. Integrative reports that combine EP findings with clinical scores and imaging results may be more readily interpretable than isolated EP parameters. Education and training of rehabilitation professionals in basic EP principles will also be critical to foster confident use.

### 8.5 Methodological heterogeneity and minimum reporting items

A major barrier to synthesizing EP evidence in stroke rehabilitation is methodological heterogeneity across stimulation parameters, recording montages, preprocessing pipelines, and endpoint definitions. For SEPs, variability in nerve selection (median vs. tibial), stimulus rate/intensity, referencing scheme, averaging strategy, and peak identification rules can materially alter latency and amplitude estimates. For MEPs, coil type/orientation, hotspot definition, thresholding method, background EMG state, and intensity calibration are frequent sources of between-study variance. ERP results are additionally sensitive to task design (stimulus probabilities, timing, instructions), trial counts retained after artifact rejection, and the a priori definition of component windows (e.g., P300 latency/amplitude quantification).

To support reproducibility and comparability—without implying clinical treatment recommendations—future studies should report a minimum set of acquisition and analysis details, ideally aligned with established methodological checklists and consensus guidance. At minimum, reports should specify: participant state and confounders (arousal level, sedatives/antiepileptics where relevant); stimulation parameters and rationale (site/nerve, intensity, rate, number of trials); recording setup (electrodes/muscles, montage/reference, impedance targets); preprocessing and artifact handling (filters, rejection/ICA rules, baseline correction for ERPs); operational definitions for EP endpoints (presence/absence criteria, latency/amplitude metrics, window definitions); and trial retention/SNR or reliability indices when feasible. Consistent reporting of these items would materially improve interpretability, enable harmonized multicenter datasets, and strengthen the evidentiary basis for translating EP biomarkers into longitudinal rehabilitation monitoring (Chipchase et al., 2012; Cruccu et al., 2008; Picton et al., 2000; Rossi et al., 2021).

## 9 Future directions and conclusion

Evoked potentials occupy a unique position among neurophysiological tools for stroke rehabilitation because they provide objective, pathway-specific and temporally precise measures that bridge microscopic neural processes with macroscopic behavior. Extensive work has established the prognostic value of MEPs, SEPs and ERPs for motor, sensory and cognitive outcomes, and more recent studies highlight their potential as dynamic biomarkers of recovery and treatment-induced plasticity. Moving forward, a key priority will be to shift from heterogeneous, single-center observational work toward more standardized and integrative frameworks. This includes harmonizing acquisition and analysis protocols across centers, developing consensus recommendations for clinical use, and embedding EP measures prospectively into well-designed rehabilitation trials. Within such frameworks, EPs can be used not only to describe recovery trajectories, but also to drive adaptive, individualized rehabilitation pathways in which therapy intensity, modality and timing are adjusted according to evolving neurophysiological profiles rather than solely on clinical scales.

At the same time, it is increasingly clear that EPs will be most powerful when considered as part of a broader multimodal biomarker ecosystem. Combining SEPs, MEPs and ERPs with structural and functional imaging, kinematic and wearable sensor data, and digital therapy platforms may offer a richer and more robust characterization of recovery than any single modality alone. Advances in portable EEG and stimulation technology, brain–computer interface paradigms, high-density recordings, and machine-learning–based data fusion are likely to accelerate this evolution and may eventually enable EP-informed monitoring in home-based and tele-rehabilitation settings. Nonetheless, important practical and conceptual challenges remain, including feasibility in routine care, interpretation in heterogeneous patient populations, and demonstration of added value over traditional assessment. In conclusion, by systematically integrating evoked potentials into stroke rehabilitation research and carefully translating these methods into clinical workflows, there is substantial potential to move from a predominantly scale-driven approach toward a more objective, mechanism-informed and personalized rehabilitation paradigm, ultimately improving functional outcomes and quality of life for stroke survivors.
